# The therapeutic engagement index in autism: a hypothesis-generating framework for stratifying intervention responsiveness and adaptive therapeutic availability

**DOI:** 10.3389/frcha.2026.1872562

**Published:** 2026-06-03

**Authors:** Yves Fuamba

**Affiliations:** Department of Autism Research, Antibiostress Clinics, Gloucester ON, Canada

**Keywords:** autism spectrum disorder, construct validation, developmental readiness, intervention responsiveness, therapeutic alliance, therapeutic engagement, translational framework, treatment heterogeneity

## Abstract

Autism spectrum disorder is characterized by substantial heterogeneity in developmental trajectories, learning profiles, therapeutic participation, and response to intervention. Although intervention type, intensity, timing, fidelity, and baseline developmental profile are important predictors of outcome, they do not fully explain why autistic children exposed to broadly comparable interventions may show markedly different patterns of participation, learning, generalization, and adaptive change. TEI is defined as a multidimensional, context-sensitive, and observable construct describing the degree to which an autistic child can access, tolerate, respond to, sustain, and generalize therapeutic input within a specific intervention context. TEI is not intended to replace established constructs such as treatment engagement, therapeutic alliance, readiness, motivation, compliance, participation, developmental readiness, or treatment response. The manuscript clarifies the conceptual novelty of TEI, distinguishes it from related constructs, proposes candidate observable domains and indicators, outlines testable hypotheses, specifies boundary conditions, and provides a staged validation roadmap. TEI is not presented as a validated scale, clinical decision tool, diagnostic measure, compliance metric, or indicator of child responsibility for treatment outcome. Rather, it is proposed as a research framework for identifying modifiable conditions of therapeutic accessibility, including regulation, support fit, relational access, context, intervention demands, and developmental state.

## Introduction

Autism spectrum disorder is marked by substantial heterogeneity in developmental course, learning profiles, therapeutic participation, and response to intervention (1–4). One of the most persistent questions in autism intervention research is why children exposed to broadly comparable therapeutic approaches may show different patterns of participation, progress, generalization, and adaptive change (5–8).

Current explanations emphasize intervention intensity, timing, setting, fidelity, baseline language, developmental level, adaptive functioning, symptom profile, family resources, and service access (5–12). These variables are essential but incomplete. They do not always explain why a child who is physically present in therapy may not be functionally available for learning, why engagement varies across contexts, or why participation does not always translate into generalizable developmental change.

Engagement-related constructs already exist in treatment research, psychotherapy, implementation science, developmental intervention, education, and child mental health (13–17). These include treatment engagement, therapeutic alliance, readiness, motivation, adherence, compliance, participation, and treatment response. However, these constructs often emphasize service use, relational collaboration, motivation, behavioral participation, or outcome rather than the specific accessibility of therapeutic input to the developing child within a given regulatory, attentional, relational, contextual, and developmental state.

This manuscript proposes the Therapeutic Engagement Index (TEI) as a hypothesis-generating translational construct for organizing this problem. TEI is not proposed as a validated clinical scale or as a replacement for established engagement constructs. Instead, it is intended to make therapeutic accessibility more observable, differentiable, and testable in autism intervention research.

The revised manuscript addresses four central questions: What is conceptually distinct about TEI? How can TEI be defined without circularity? What observable domains and indicators could support future operationalization? Under what conditions should TEI be applied, tested, or limited? The goal is to move TEI from a broad conceptual label toward a falsifiable research construct. Table 1 introduces the contrast between TEI and related constructs.

**Table 1:** Distinguishing TEI from related constructs.

Construct	Primary focus	Typical unit of observation	Limitation for explaining autism intervention responsiveness	How TEI differs
Treatment engagement	Involvement in treatment process or service use	Attendance, participation, involvement, homework, session behavior	May emphasize service use or participation without specifying accessibility to therapeutic learning	TEI focuses on functional access, tolerance, response, sustained effort, and generalization within a specific intervention context
Therapeutic alliance	Collaborative relationship and bond with therapist	Therapist-child or therapist-family relationship, agreement on tasks/goals	Relationally important but does not fully capture regulatory, attentional, or developmental accessibility	TEI includes relational-motivational access as one domain but also includes regulation, attention, responsiveness, persistence, and generalization
Readiness	Preparedness for change or intervention	Global readiness, motivation, stage of change, developmental readiness	May be too broad or static and often not specific to session-level therapeutic input	TEI is context-sensitive and can vary across sessions, tasks, supports, and environments
Motivation	Interest, willingness, or drive to participate	Preference, affect, reward sensitivity, task interest	May misattribute low engagement to lack of desire while ignoring regulation, sensory load, fatigue, or support fit	TEI treats motivation as relational-contextual and only one contributor to therapeutic accessibility
Compliance/adherence	Following instructions or completing prescribed activities	Instruction following, protocol completion, session attendance	Can be child-blaming and may confuse obedience with learning accessibility	TEI explicitly rejects compliance as the core construct and focuses on therapeutic access and support conditions
Participation	Observable involvement in activities	Presence, task involvement, social participation	Visible participation may occur without deep learning, generalization, or regulation-supported access	TEI includes participation but requires responsiveness, persistence, and generalization indicators
Treatment response	Change following intervention	Outcome measures, symptom change, skill gains	Outcome does not explain process and may be measured after intervention effects occur	TEI is proposed as an intermediate process construct that may help explain response
Therapeutic engagement index	Functional accessibility of therapeutic input	Observable domains across regulation, attention, support response, persistence, transfer, and relational-motivational engagement	Not yet validated; requires operationalization and testing	TEI integrates multiple process domains into a testable framework for intervention responsiveness research

## Conceptual novelty and unique contribution of TEI

The unique contribution of TEI is not the general claim that engagement matters. That claim is already well supported across intervention, psychotherapy, education, and developmental literatures (13–17). The proposed contribution is narrower: TEI conceptualizes engagement as the functional accessibility of therapeutic input to an autistic child within a specific intervention context.

TEI differs from treatment attendance or adherence because it is not primarily concerned with whether the child is present or whether sessions are completed. It differs from compliance because it does not evaluate obedience or cooperation. It differs from therapeutic alliance because it is not centered mainly on the relational bond between therapist and client or family, although relational access may influence TEI (13, 14, 17). It differs from readiness models because it is not a global readiness-to-change construct. It differs from motivation because it does not reduce engagement to desire, interest, or willingness. It differs from participation because visible participation may occur without deep regulatory, cognitive, or developmental access to therapeutic learning. Finally, TEI differs from treatment response because it is proposed as a process construct that may help explain response, not as the response itself.

TEI is therefore positioned as an intermediate translational construct between intervention exposure and intervention responsiveness. It asks whether the child can access, tolerate, process, sustain, and generalize therapeutic input under current regulatory, attentional, relational, contextual, and developmental conditions. Table 1 contrasts TEI with treatment engagement, therapeutic alliance, readiness, motivation, compliance/adherence, participation, developmental readiness, and treatment response.

## Definition of the therapeutic engagement index

TEI is defined as a multidimensional, context-sensitive, and observable construct describing the degree to which an autistic child can access, tolerate, respond to, sustain, and generalize therapeutic input within a specific intervention context.

This definition intentionally avoids defining TEI by outcome alone. A child may respond well to an intervention because TEI is high, but TEI itself should be measured through observable process indicators before the outcome is known. Similarly, a child may show low visible participation because the task is poorly matched, the environment is overwhelming, the therapeutic relationship is not yet secure, or the child is experiencing fatigue, stress, pain, dysregulation, or contextual overload. TEI should therefore be interpreted as a relational and contextual accessibility construct rather than as a child-blaming trait.

TEI has six candidate domains: regulatory availability; attentional and interactional access; responsiveness to therapeutic support; persistence and adaptive effort; contextual transfer and generalization; and relational-motivational engagement. These domains are analytically separable but expected to interact in real-world intervention settings. Table 2 provides the candidate operationalization framework for these six TEI domains, including observable indicators, measurement sources, provisional scoring logic, and validation requirements.

**Table 2:** Candidate TEI domains, observable indicators, measurement sources, and validation requirements.

TEI domain	Definition	Observable indicators	Measurement sources	Provisional scoring logic	Validation requirement
Regulatory availability	Capacity to remain sufficiently organized and recoverable to access therapeutic input	Calm-alert state, transition tolerance, recovery after distress, co-regulation need, arousal stability	Session observation, clinician rating, caregiver report, optional physiological data	Domain rating across sessions; low/high uncertainty flags	Inter-rater reliability; sensitivity to state change
Attentional and interactional access	Capacity to orient to people, materials, cues, or shared activities	Response to cues, joint attention, attention shifting, shared activity tolerance, interactional availability	Structured observation, therapist logs, caregiver report	Context-specific rating; repeated measures preferred	Convergent validity with observed engagement
Responsiveness to therapeutic support	Capacity to benefit from scaffolding, prompting, modeling, reinforcement, or co-regulation	Response to support, reduced distress with adjustment, improved task access after scaffolding	Therapist rating, session coding, intervention records	Support-response profile rather than compliance score	Predictive validity for proximal learning
Persistence and adaptive effort	Capacity to sustain or re-engage during challenge	Task persistence, re-engagement after error, frustration tolerance, adaptive help-seeking	Observation, caregiver/clinician ratings, longitudinal logs	State-sensitive effort profile	Reliability and sensitivity to intervention/context changes
Contextual transfer and generalization	Capacity to carry engagement or learning across contexts	Skill use across settings, people, routines, materials; reduced context dependence	Caregiver report, school/clinic reports, direct observation	Cross-context transfer rating	Longitudinal validation; ecological validity
Relational-motivational engagement	Degree to which relational safety, autonomy support, affective access, and meaningfulness support therapeutic engagement	Interest, positive affect, trust, relational access, preference, meaningful participation	Observation, therapist/caregiver report, child input when feasible	Relational-contextual profile; not a motivation blame score	Stakeholder validity; cultural/contextual validation

## Candidate TEI domains

### Regulatory availability

Regulatory availability refers to the child's observable capacity to remain sufficiently organized, calm-alert, and recoverable to access therapeutic input. Candidate indicators include tolerance of transitions, recovery after frustration, regulation of arousal, ability to remain available without escalating distress, and need for co-regulation. This domain is consistent with intervention research emphasizing regulation, participation, and child process factors as contributors to therapeutic outcomes (5, 11, 13, 14).

### Attentional and interactional access

Attentional and interactional access refers to the child's ability to orient to people, materials, cues, or shared activities in ways that allow intervention input to become usable. Indicators may include response to name or cues, joint attention availability, visual or auditory orientation, shared activity tolerance, and ability to shift attention without excessive distress. This domain is relevant to social-communication interventions and joint-attention approaches (15, 16).

### Responsiveness to therapeutic support

Responsiveness to therapeutic support refers to the child's observable response to scaffolding, prompting, modeling, reinforcement, environmental adjustment, relational support, or co-regulation. It is not equivalent to compliance; rather, it reflects whether the support structure makes therapeutic input more accessible. This domain is relevant to research on treatment process, intervention fit, and predictors of intervention response (5, 6, 8, 11, 13, 14).

### Persistence and adaptive effort

Persistence and adaptive effort refer to the child's capacity to continue engaging with a task or interaction despite challenge, novelty, or partial failure. Indicators may include task persistence, recovery after error, frustration tolerance, adaptive help-seeking, and willingness to re-engage after support. This domain may help explain why exposure alone does not fully account for treatment gains (7, 9, 10, 12).

### Contextual transfer and generalization

Contextual transfer and generalization refer to whether engagement and learning can move beyond the immediate therapeutic setting. Indicators may include use of learned skills across people, settings, materials, or routines, and whether engagement is maintained when support conditions change. This domain is particularly important because intervention effects may vary across contexts and outcome measures (4, 6, 7).

### Relational-motivational engagement

Relational-motivational engagement refers to the degree to which relational safety, meaningfulness, affective connection, and interest support therapeutic access. It includes motivation but is broader than motivation alone because it depends on relational fit, context, autonomy support, predictability, and perceived relevance. This domain overlaps with therapeutic process research while remaining embedded within a broader TEI profile (13, 14, 17).

## Causal framework and domain interaction mechanisms

The proposed causal framework should be interpreted as a provisional hypothesis rather than as an established pathway. Upstream conditions such as regulatory burden, sensory load, sleep, fatigue, pain, stress, environmental predictability, therapeutic fit, caregiver/clinician support, and developmental level may influence one or more TEI domains. TEI domains may then influence how therapeutic input is processed, retained, generalized, and translated into adaptive change.

**Figure 1:**
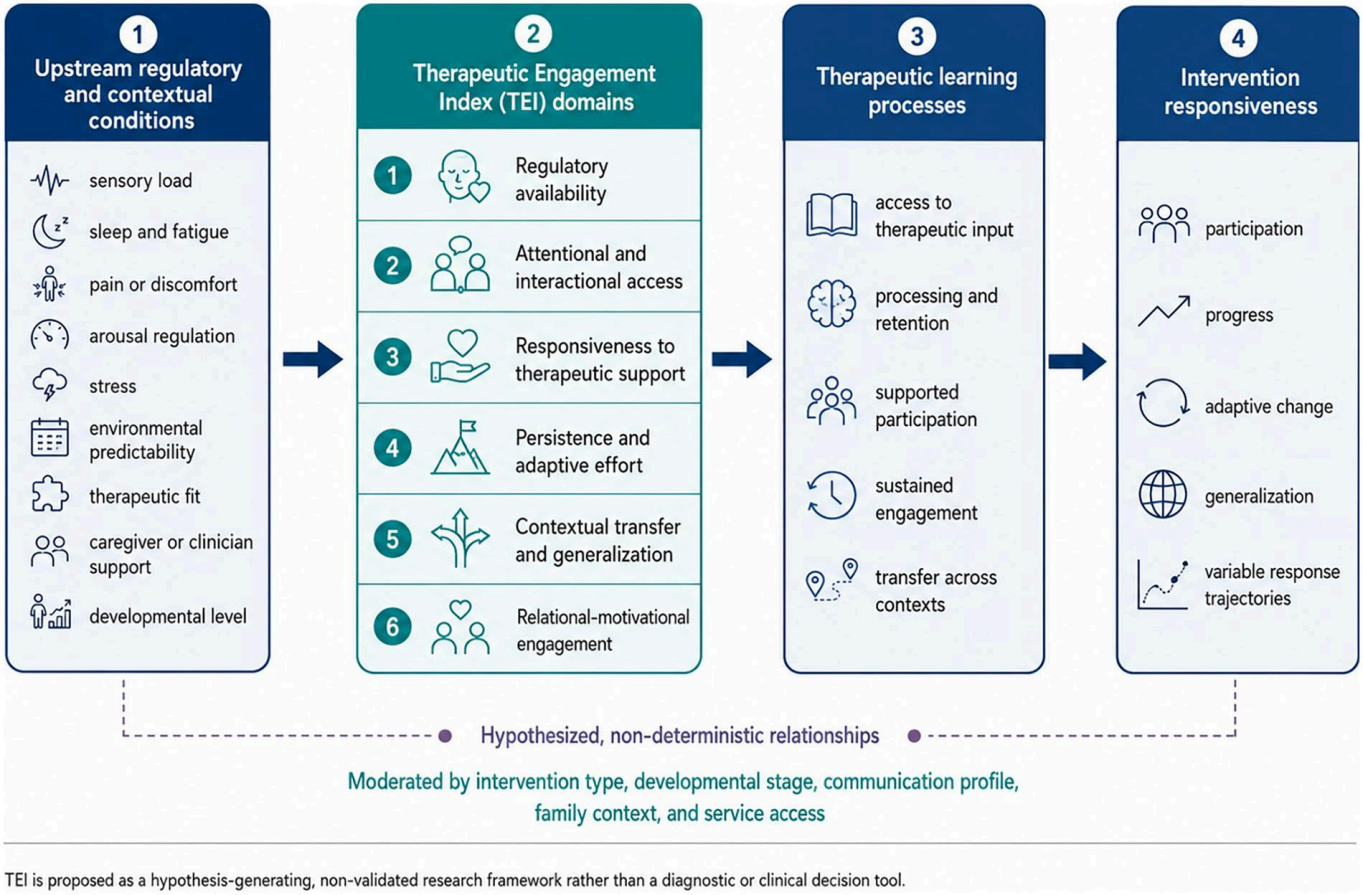
Proposed causal framework for the therapeutic engagement Index in autism. The figure illustrates TEI as a hypothesis-generating process construct situated between upstream regulatory/contextual conditions and intervention responsiveness. Upstream conditions may include sensory load, sleep, fatigue, pain, arousal regulation, environmental predictability, therapeutic fit, relational safety, caregiver/clinician support, and developmental level. These conditions may influence TEI domains, including regulatory availability, attentional and interactional access, responsiveness to support, persistence and adaptive effort, contextual transfer and generalization, and relational-motivational engagement. TEI domains are proposed to influence therapeutic learning processes and intervention responsiveness. The model is not a validated causal pathway and should not be used as a diagnostic, compliance, or treatment-selection tool.

This framework can be expressed as: upstream regulatory/contextual conditions → TEI domains → therapeutic learning processes → intervention responsiveness. The arrows do not imply deterministic causation. Relationships may be reciprocal, context-dependent, and moderated by intervention type, developmental stage, communication profile, intellectual/developmental disability, family context, and service access. Figure 1 summarizes this provisional causal framework.

Parsimony is important. TEI should not become a container for every factor affecting intervention outcome. Its core function is to represent therapeutic accessibility. Variables such as symptom severity, baseline language, adaptive functioning, intervention intensity, treatment fidelity, family resources, and service access should be modeled as covariates, moderators, or contextual conditions rather than automatically included within TEI itself (5–12).

## Testable hypotheses

TEI should be evaluated through explicit hypotheses rather than assumed clinical intuition. The following hypotheses are proposed for future empirical work and are grounded in prior literature on predictors, process factors, and intervention-response heterogeneity (5–14, 18, 19):

Hypothesis 1: TEI domain scores will predict intervention responsiveness beyond intervention exposure alone.

Hypothesis 2: TEI will add incremental predictive value beyond established predictors such as age, baseline language level, adaptive functioning, symptom severity, intervention intensity, treatment fidelity, and family/service context.

Hypothesis 3: Regulatory availability and attentional-interactional access will show stronger associations with early session participation, whereas contextual transfer and generalization will show stronger associations with longer-term functional outcomes.

Hypothesis 4: TEI will vary across time and context, showing state sensitivity rather than functioning as a fixed trait.

Hypothesis 5: Children with similar baseline severity but divergent intervention trajectories will differ in TEI profiles.

Hypothesis 6: Changes in TEI over time will precede or accompany changes in intervention responsiveness.

Hypothesis 7: TEI will mediate or moderate associations between upstream regulatory/contextual burden and intervention responsiveness.

These hypotheses are falsifiable. TEI would be weakened as a construct if it fails to show reliable measurement, fails to predict responsiveness beyond standard predictors, overlaps entirely with existing engagement constructs, or cannot be distinguished from treatment response itself. These hypotheses also define the staged validation logic summarized in Table 3.

**Table 3:** Staged validation roadmap for TEI.

Validation stage	Core question	Candidate methods	Minimum evidence needed
Content validity	Are domains meaningful, observable, and non-stigmatizing?	Expert review, caregiver input, autistic stakeholder consultation, construct mapping	Clear domains and acceptable terminology
Item/domain development	Can domains be translated into measurable indicators?	Item generation, session coding, caregiver/clinician rating design, feasibility review	Low-burden, observable candidate items
Reliability	Can TEI indicators be measured consistently?	Inter-rater reliability, caregiver-clinician convergence, test-retest where appropriate	Reliable ratings while preserving state sensitivity
Construct validity	Is TEI distinct from related constructs?	Convergent/discriminant analyses with engagement, alliance, compliance, symptom severity, readiness	Related but non-redundant construct structure
Longitudinal validity	Does TEI change meaningfully over time and context?	Repeated measures, trajectory modeling, session-to-session tracking	Meaningful state-sensitive variation
Predictive validity	Does TEI predict intervention responsiveness?	Prospective studies, outcome association, mediation/moderation analysis	Prediction beyond exposure and baseline profile
Incremental validity	Does TEI add value beyond standard predictors?	Regression or machine-learning models including age, language, adaptive function, severity, intensity, fidelity	Improved explanatory or predictive performance
Cross-context and fairness validation	Does TEI generalize across contexts and populations?	Multi-site, cross-cultural, intervention-type, language, age, ID/DD subgroup analyses	No systematic bias or inequitable interpretation
Clinical utility assessment	Would validated TEI improve formulation or support planning?	Prospective feasibility and usability studies, ethical review	Only after reliability, validity, fairness, and feasibility are established

## Boundary conditions

TEI should be applied only under defined conditions. First, there must be an identifiable therapeutic, educational, developmental, or support context in which engagement can be observed. Second, intervention demands and supports must be described. Third, at least some indicators should be observable or reportable across sessions or contexts. Fourth, TEI should be interpreted alongside baseline developmental profile, communication level, adaptive functioning, intervention fidelity, environmental demands, and family/service context. Table 4 summarizes key boundary conditions and safeguards for TEI interpretation.

**Table 4:** Boundary conditions and safeguards for TEI interpretation.

Boundary or safeguard	Appropriate interpretation	Inappropriate interpretation to avoid
Use context	TEI is used in identifiable therapeutic, educational, developmental, or support contexts	Using TEI as a general autism severity score
Construct meaning	TEI reflects therapeutic accessibility under documented support and context conditions	Treating TEI as motivation, compliance, or child responsibility
Measurement design	Repeated observation and multi-informant data are preferred	Inferring stable traits from one session
Clinical status	TEI is hypothesis-generating until validated	Using TEI for diagnosis, treatment selection, or service eligibility
Ethical use	Low TEI prompts support-fit, regulation, context, sensory, fatigue, pain, and relational questions	Blaming the child or family for limited progress
Equity	Service access, cultural context, language, and resource differences must be considered	Comparing children without accounting for systemic and contextual barriers

TEI should not be used as a diagnostic measure, global severity score, compliance rating, motivation judgment, eligibility criterion, or proxy for treatment effectiveness. It should not be used to blame the child for limited progress. A low TEI profile should prompt investigation of modifiable conditions: regulation, sensory load, task fit, relational safety, support intensity, environmental predictability, pacing, communication access, fatigue, pain, caregiver stress, and intervention demands.

TEI may be most useful in longitudinal or repeated-measure designs. A single session rating may be insufficient because engagement is state-sensitive and context-dependent. Boundary conditions should therefore be built into future measurement systems before TEI is interpreted as a stable profile.

## Validation roadmap

A staged validation roadmap is required before TEI can be treated as more than a conceptual framework. The roadmap summarized in Table 3 begins with content validity. Clinicians, researchers, caregivers, and autistic stakeholders should evaluate whether candidate TEI domains are meaningful, non-stigmatizing, observable, and distinct from related constructs.

The second stage is item and domain development. Candidate indicators should be translated into structured observation items, caregiver-report items, clinician ratings, or session-coding variables. Items should be tested for clarity, burden, redundancy, and feasibility.

The third stage is reliability testing. This includes inter-rater reliability for observational ratings, caregiver-clinician convergence, test-retest stability where appropriate, and sensitivity to state changes. Because TEI is expected to vary across contexts, excessive stability is not necessarily the goal; rather, the construct should distinguish meaningful state-sensitive variation from measurement noise.

The fourth stage is construct validity. TEI should show convergent associations with relevant engagement and process measures while remaining distinguishable from alliance, compliance, attendance, symptom severity, and treatment outcome. The fifth stage is longitudinal and predictive validation: TEI should be tested as a predictor, mediator, or moderator of intervention responsiveness. The sixth stage is incremental validity: TEI should improve explanatory or predictive models beyond established predictors (5–8, 11, 12, 19).

The seventh stage is cross-context, cross-cultural, and developmental validation. TEI indicators may differ across language, culture, intervention model, communication profile, age, and support context. Validation must therefore examine fairness, feasibility, and bias before any clinical translation is considered. Table 3 summarizes this roadmap.

## Ethical safeguards and interpretive cautions

TEI must be developed with safeguards against misinterpretation. A central ethical risk is that low therapeutic engagement could be interpreted as lack of motivation, poor cooperation, family failure, or child responsibility for limited progress. The framework explicitly rejects this interpretation. Low TEI should be understood as a signal that therapeutic accessibility conditions may not be adequately aligned with the child's current regulatory, relational, developmental, sensory, contextual, or support needs.

The construct should also avoid reinforcing inequities. Families with fewer resources may have less access to high-quality intervention, medical evaluation, stable services, or supportive environments. A low TEI profile in such contexts could reflect system-level barriers rather than child-level limitations. Future TEI research should therefore measure service access, environmental predictability, family stress, intervention quality, and cultural context.

TEI should also avoid over-medicalization. Physiological or regulatory factors may influence engagement, but TEI is not a biomarker test and should not be used to justify unsupported biological interventions (20). Its immediate purpose is to structure observation and research, not to prescribe treatment.

## Discussion

The TEI framework addresses a clinically important gap: therapeutic exposure alone does not fully explain intervention responsiveness in autism (5–12). The revised framework proposes TEI as an intermediate, observable, and context-sensitive construct that may help explain how therapeutic input becomes accessible or inaccessible to a child at a given time.

The framework's theoretical value depends on its differentiation from existing constructs. Treatment engagement, therapeutic alliance, readiness, motivation, participation, adherence, and intervention response all capture important aspects of therapeutic process (13, 14, 17). TEI is intended to integrate but not duplicate these constructs by focusing on functional accessibility to therapeutic learning. Its empirical value will depend on whether this distinction can be measured and validated.

The framework also emphasizes parsimony. TEI should not absorb all predictors of intervention outcome. It should remain focused on the child's observable therapeutic accessibility within a documented intervention context, while baseline developmental profile, intervention fidelity, family context, service access, and cultural factors are modeled separately.

At its strongest, TEI may help researchers identify why children with similar baseline profiles and intervention exposure show different response trajectories. At its weakest, TEI could become a broad label for engagement without added explanatory value. The validation roadmap is therefore essential. TEI must be tested against related constructs, standard predictors, and longitudinal outcomes before translational use is inferred.

## Limitations

This manuscript remains conceptual. It does not present original data, a validated TEI scale, psychometric testing, clinical trial findings, biomarker validation, or predictive models.

TEI domains are candidate domains rather than final measurement domains. Their boundaries may overlap, and empirical work may show that some domains should be combined, subdivided, or removed.

The causal framework is provisional. Relationships among regulation, attention, support responsiveness, persistence, generalization, relational engagement, and intervention responsiveness may be bidirectional, moderated by context, or confounded by developmental level, family resources, treatment fidelity, and service access.

The framework may not apply equally across intervention types, ages, communication profiles, intellectual/developmental disability levels, cultures, or service settings. Cross-context and cross-cultural validation will therefore be required.

Finally, TEI should not be used clinically until reliability, validity, fairness, feasibility, and utility are demonstrated. It is not a diagnostic tool, compliance metric, child-blaming label, or treatment-selection instrument.

## Conclusion

The Therapeutic Engagement Index is proposed as a hypothesis-generating translational construct for studying heterogeneity in intervention responsiveness in autism. TEI is defined as the degree to which an autistic child can access, tolerate, respond to, sustain, and generalize therapeutic input within a specific intervention context.

The framework's unique contribution is to position therapeutic engagement as a multidimensional accessibility construct situated between intervention exposure and intervention responsiveness. By distinguishing TEI from alliance, readiness, motivation, compliance, participation, and treatment response, the manuscript provides a more precise starting point for empirical testing.

Future research should focus on operationalization, measurement development, reliability, construct validity, longitudinal testing, predictive and incremental validity, boundary conditions, and ethical safeguards. Until these steps are completed, TEI should be regarded as a conceptual research framework rather than a validated clinical tool.

## Data Availability

The original contributions presented in the study are included in the article/Supplementary Material, further inquiries can be directed to the corresponding author.
